# Intestinal Parasitic Infections, Treatment and Associated Factors among Pregnant Women in Sao Tome and Principe: A Cross-Sectional Study

**DOI:** 10.1155/2022/7492020

**Published:** 2022-11-18

**Authors:** Alexandra Vasconcelos, Swasilanne Sousa, Nelson Bandeira, Marta Alves, Ana Luísa Papoila, Filomena Pereira, Maria Céu Machado

**Affiliations:** ^1^Unidade de Clínica Tropical - Global Health and Tropical Medicine (GHTM), Instituto de Higiene e Medicina Tropical (IHMT), Universidade NOVA de Lisboa, Lisboa, Portugal; ^2^Hospital Dr. Ayres de Menezes, São Tomé, Sao Tome and Principe; ^3^CEAUL, NOVA Medical School/Faculdade de Ciências Médicas, Universidade Nova de Lisboa, Lisboa, Portugal; ^4^Faculdade de Medicina de Lisboa, Universidade de Lisboa, Lisboa, Portugal

## Abstract

**Background:**

Intestinal parasitic infections (IPIs) are a public health problem in developing countries such as Sao Tome and Principe (STP) although the pregnancy burden of IPIs is unknown in this endemic country. Thus, the aim of this study was to determine the prevalence of IPIs, prescribed anthelmintics, and associated factors among pregnant women admitted to Hospital Dr. Ayres de Menezes (HAM).

**Methods:**

A hospital-based cross-sectional study was conducted among pregnant women admitted to the HAM who had undergone antenatal copro-parasitological screening. Data were abstracted from antenatal care (ANC) cards regarding parasitological results and anthelmintic prescriptions. A structured questionnaire face-to-face interview was also applied. Pregnant women with an IPI (210) were compared to noninfected women (151). Data analysis was performed using SPSS version 25.0. Odds ratios (ORs) with 95% confidence intervals (CIs) for factors associated with IPIs were estimated using multiple logistic regression models. A *p* value <0.05 was considered statistically significant.

**Results:**

A total of 361 participants (210 IPI and 151 no-IPI) with a mean age of 26.96 (SD: 7.00) were included. The overall prevalence of IPI was 58.2% (95% CI 52.9 to 63.3), mainly due to helminthiasis, with a 55.9% (95% CI 50.7–61.2%) rate. *Ascaris lumbricoides* (90.9%) was the most predominant parasite species identified followed by *Trichuris trichiura* (13.8%). Polyparasitism was observed in 25 cases (11.9%). Anthelmintics were prescribed to 23% of pregnant women. *S intercalatum* (11) and *E histolytica* (7) infections were not adequately treated. IPI was significantly associated with primary education (AOR 1.73 (95% CI: 1.10–2.71)), unemployment (AOR 1.94 (95% CI: 1.20–3.13)), and parity of five or above (AOR 3.82 (95% CI: 1.32–11.08)).

**Conclusion:**

This study highlights the IPI burden, associated factors, and missing treatment opportunities among pregnant women with STP. This study is a useful tool for policymakers in STP to enhance the health of women and their unborn babies.

## 1. Introduction

Intestinal parasitic infections (IPIs) are neglected tropical diseases (NTDs) and a burden to pregnant women, with a prevalence that ranges from 24 to 70% in low- and medium-income countries (LMICs), with rates of coinfection with different parasites (polyparasitism) of approximately 10% [1]. Globally, among pregnant women, the most common infestation is hookworm and whipworm (*Trichuris trichiura*), followed by *Ascaris lumbricoides* [1–4].

Less frequent but also prone to cause adverse birth outcomes and complications during pregnancy are invasive enteric parasites such as Schistosoma *spp*. and the protozoan *Entamoeba histolytica* [1]. Schistosomiasis affects approximately 200 million people and is “second only to malaria in public health importance,” with pregnant women being one of the important at-risk groups [5, 6]. *Entamoeba histolytica*, responsible for amebiasis, remains one of the top three parasitic causes of mortality, with an estimated 50 million people affected worldwide [7, 8].

The major concerns regarding IPI in pregnant women are the associated higher risks for adverse maternal outcomes, such as anemia as well as poor pregnancy weight gain [9–11]. Additionally, IPI implications in newborn health are the risk of low birth weight (LBW), intrauterine growth retardation, prematurity, stillbirths, and neonatal mortality, a considerable burden in countries that already have a high rate of neonatal mortality and morbidity [12–14].

Regarding the anthelmintic treatment of IPI during pregnancy, there is still some controversy [15, 16], although it is endorsed by the World Health Organization (WHO) [17–19]. The most recommended drugs for treating IPI in pregnancy are albendazole for helminths, praziquantel for Schistosoma, and metronidazole for protozoan parasites [20, 21]. Nonetheless, available evidence shows that most pregnant women in IPI-endemic settings do not receive any deworming medication, with the WHO setting a target of reaching a 75% rate of deworming pregnant women by 2030 [3, 22].

Knowing each country's reality and specific associated factors promoting IPI among pregnant women will allow us to tackle it more accurately. In general, factors associated with IPIs are intimately connected with poverty and are the same for both the general population and pregnant women [23]. For instance, a well-known independent risk factor is WASH, an acronym for inadequate water supply, sanitation, and hygiene, which is also the major factor responsible for mortality and burden of disease due to diarrhea-causing infections in LMICs [24, 25].

Sao Tome and Principe (STP) is the second smallest African country, a low-income setting, endemic for helminthiasis and schistosomiasis (*S intercalatum*) [26, 27]. The current country strategy is preventive IPI chemotherapy to be given only to preschool and school-aged children [28]. Infected pregnant women receive anthelmintic treatment in the second or third trimester on a case-by-case basis although the country's prevalence of anemia among pregnant women is around 61% [29].

To our knowledge, intestinal parasitic infections among pregnant women in Sao Tome and Principe have never been studied. This study was undertaken within the context of a broader project on the causes and risk factors contributing to neonatal mortality and adverse birth outcomes in STP [30, 31].

We sought to determine the prevalence of IPIs, prescribed anthelmintics, and associated factors among pregnant women admitted to Hospital Dr. Ayres de Menezes.

Thus, these research findings will help to design targeted interventions for these NTDs among pregnant women in STP and better allocate resources to where they are needed.

## 2. Materials and Methods

### 2.1. Study Design

A hospital-based cross-sectional study was carried out on pregnant women admitted to the HAM maternity unit for delivery.

### 2.2. Setting

The archipelago of Sao Tome and Principe is an African island nation, with 219,161 inhabitants [29]. The level of poverty is high, with 47% of the population practicing open defecation and only 69.8% having access to clean and safe drinking water [29].

This study was conducted at the HAM maternity unit, which has an annual average delivery rate of 4500 births, approximately 82.4% of all deliveries in the country, as it is the only hospital in the country. Antenatal care (ANC) coverage in STP is high, and women are asked to perform a routine copro-parasitological exam during the pregnancy period. The exam consists of stool sampling using the direct wet mount method and formol-ether concentration method and then observation by trained parasitologists who declare it positive when various stages of the parasites, such as trophozoites, cysts, ova, and larvae, are identified. The results from copro-parasitological exams, as well as prescribed anthelminthics, are registered by the nurses in the ANC pregnancy card that pregnant women carry along all ANC visits and bring to the HAM at the time of labor.

### 2.3. Participants

All pregnant women admitted to the HAM maternity unit for delivery constituted the source population, whereas the study populations were selected pregnant women admitted to the HAM maternity unit during the study period. The recruitment occurred from July 2016 to November 2018.

### 2.4. Eligibility Criteria

All women admitted to the HAM maternity unit for delivery with a gestational age of 28 weeks or more were eligible to be enrolled in the study. Those who gave birth in the rural basic maternity units but were immediately transferred to HAM for postpartum medical evaluation or newborn follow-up were also eligible to be included in the study.

The exclusion criteria included the following: (1) pregnant women with gestational age less than 28 weeks, (2) women who had no ANC pregnancy card, (3) women with cognitive impairment, (4) women who were unstable due to postpartum complications, and (5) adolescent or illiterate mothers who had not obtained permission from their parents or legal guardians to participate in the study. Pregnant women without ANC copro-parasitological screening (147) and pregnant women with HIV, sickle cell disorder, and malaria (10) were also excluded from this study.

**Figure 1:**
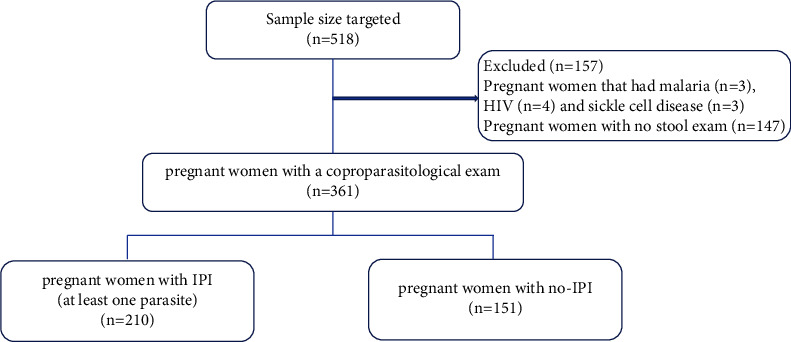
Flowchart of participation in the study.

A total of 361 pregnant women with a copro-parasitological exam performed during the ANC period and with the result registered in the pregnant ANC card were included. Pregnant women were divided into two groups, pregnant women with intestinal parasite infection (at least one) versus those without any IPI for associated factor assessment. A flowchart of participation in the study is shown in Figure 1.

### 2.5. Sampling Method

The software used for sample calculation was Raosoft (http://www.raosoft.com/samplesize.html), and a minimum sample size of *S* = 355 was recommended. For the original study, participants were enrolled based on the following assumptions: a two-sided 95% confidence level and a power of 80% to detect an odds ratio of at least two for neonatal adverse birth outcomes. Since the sample size was not calculated for the present outcomes, a power analysis was performed, varying from 79% to 89% for this study.

Pregnant women were selected randomly until the required sample size was achieved. Participants were invited to participate in the study after admission to the maternity unit of HAM. Each day, from the pile of admission folders, every second interval folder was selected and then carried on asking for consent for enrollment. Women's consent to participate in the study was obtained at the time of admission at HAM, but the interview was held after a woman was stabilized and ready to be discharged.

### 2.6. Data Source

The data source for this study consisted of data abstracted from the ANC pregnancy card plus a face-to-face interview. The ANC pregnancy card of each study participant was used to collect information such as the copro-parasitological result and anthelmintic prescription. Data on the sociodemographic characteristics of the participants were gathered using a structured questionnaire through a face-to-face interview with the main investigator before the women were discharged. Issues covered included the mother's and father's socioeconomic characteristics and reproductive history. All data were recorded in the app survey tool.

### 2.7. Study Variables

The exposure variables in this study include major parasitic infections as recorded in the ANC pregnancy card, such as helminthiasis (*Ascaris lumbricoides*, *Trichuris trichiura*, *Ancylostoma duodenale*, and *Strongyloides stercoralis*), schistosomiasis (*Schistosoma intercalatum*), and protozoans as amebiasis (*Entamoeba histolytica*).

Polyparasitism was defined as the presence of two or more parasite species in the same host.

The sociodemographic variables studied were as follows: (i) maternal age, (ii) maternal education, (iii) maternal occupation, (iv) marital status, (v) number of antenatal care visits, and (vi) area of residence. Mother occupation variable was categorized into two groups: employed (professional and service) and unemployed (housewife, farmer, business, and student). Education was included as a categorical variable: no education, primary education, secondary education, and higher education. The residence was defined as urban or rural, the first when women were living in the capital city (Água Grande) and rural in all other districts. We also analyzed hygiene and sanitation variables such as having access to improved water and type of toilet (toilets with flush and pit latrines) or open defecation. Parity was categorized as nullipara (0), multipara (1–4), and grand multipara (5 or above).

### 2.8. Data Quality Control

The questionnaires were administered in Portuguese, the national language. Continuous follow-up and supervision of data collection were made by the supervisors. The collected data were checked daily for completeness. The field investigator (a pediatrician) executed and was responsible for the main activities as follows: (1) obtaining consent and enrollment of the mothers, (2) face-to-face interviews, (3) abstraction of data from antenatal pregnancy cards, and (4) data entry into the app survey tool.

### 2.9. Statistical Analyses

Data analysis was carried out in three stages. The first stage involved pooling data for descriptive statistics with categorical variables being presented as frequencies and percentages and quantitative variables as the mean and standard deviation. In the second stage, the chi-square test and Fisher's exact test were used to describe the relationship between pregnant women with IPI and the categorical explanatory variables. The third stage involved a univariable analysis to identify the candidate variables for the multivariable analysis (with a *p* value <0.25). In all these analyses, logistic regression models were applied. Linearity in the logit assumption of age was verified with generalized additive regression models. Crude odds ratios (CORs) and adjusted odds ratios (AORs) with corresponding 95% confidence intervals (95% CI) were estimated. The level of significance *α* = 0.05 was considered. All data were entered into QuickTapSurvey (2010–2021 Formstack) and further analyzed using the Statistical Package for the Social Sciences for Windows, version 25.0 (IBM Corp. Released 2017. IBM SPSS Statistics for Windows, Version 25.0. Armonk, NY: IBM Corp.).

### 2.10. Ethics Approval and Consent to Participate

The study complies with the Declaration of Helsinki and was approved and consented to by the Ministry of Health of Sao Tome and Principe and by the main board of Hospital Dr. Ayres de Menezes since at the time the study protocol was submitted that there was no ethics committee in STP. Only recently has the country's National Ethics Committee been appointed. Previously, study analysis and approval were performed by dedicated ethics oversight bodies, such as the Ministry of Health of Sao Tome and Principe and the main board of the Hospital Dr. Ayres de Menezes, and both approved this study.

Moreover, all methods in our study were performed in accordance with the relevant guidelines and regulations in practice.

Written informed consent was obtained from all participants after the purpose of the research was explained orally by the researcher. Approval by the participants' parents or legal guardians was asked in the case of adolescents under 16 years of age or illiterate women.

The participants or their legal representatives also consented to have the results of this research work to be published. Participation in the survey was voluntary, as participants could decline to participate at any time during the study.

## 3. Results

### 3.1. Characteristics of the Population

A total of 361 pregnant women with a mean age of 26.96 (SD: 7.00) years old were included in this study.

Most of the participants had primary education, lived in rural areas, and were unemployed as described in Table 1. More than three-quarters of the participants were multiparous, and half had complete ANC attendance. A high proportion had access to improved water sources, and half of the pregnant women had sanitation.

### 3.2. Prevalence of Parasitic Infections

Of the total number of pregnant women, 210 (58.2%, 95% CI 52.9 to 63.3) were infected with at least one pathogenic parasite. The types of parasites identified in the 210 copro-parasitological specimens are presented in Table 2.

Coinfection with different parasites (polyparasitism) in the same host was observed in 25 pregnant women (11.9%), as described in Table 3.

Helminthiasis in monoparasitic infections (with *Ascaris lumbricoides*, *Trichuris trichiura*, *Ancylostoma duodenale*, or *Strongyloides stercoralis*) was found in 202 pregnant women, with a prevalence of 55.9% (95% CI: 50.7 to 61.2).

Schistosomiasis (*S intercalatum*) was found in 3.0% (11/361) (95% CI: 1.5 to 5.4), and amebiasis with the protozoan *E histolytica* was found in 1.9% (7/361) (95% CI: 0.8 to 3.9), including polyparasitic infections.

Regarding *S intercalatum* infection (*n* = 11), 72.7% (8) were aged 20–34 years old, 72.7% (8) had primary education, 100% (11) were married/union, 90.9% (10) had access to improved water, 63.6% (7) were living in an urban area, 54.5% (6) had sanitation, 54.5% (6) were multiparous, and 72.7% (8) had a completed ANC (8 or above visits).

### 3.3. IPI Pregnant Women Treatment

The anthelminthic prescriptions registered in the ANC pregnancy cards were albendazole, mebendazole, metronidazole, and piperazine.  Table 4 further describes the prescription according to the type of parasite. Regarding the helminthiasis group, a total of 23.8% (46) received anthelminthic treatment. Adequate treatment prescription (albendazole, mebendazole, or piperazine) was identified in 40 pregnant women with helminthic-IPI. Five received metronidazole inadequately.

Anthelminthic prescriptions for women infected with *S intercalatum* and *E histolytica* were all identified as inadequate (Table 4).

### 3.4. Pregnant Women with IPI versus Pregnant Women with No-IPI

The comparison of sociodemographic characteristics between pregnant women with IPIs and those without IPIs is shown in Table 1.

### 3.5. Factors Associated with IPIs in Pregnant Women: Univariable Analysis

Table 5 concerns the univariable regression analysis to identify candidates for the multivariable analysis among factors for IPI during pregnancy. The results indicate that the odds of having an IPI during pregnancy increased twice for pregnant women with primary education (cOR 2.58; 95% CI 1.14–5.83; *p* = 0.023). The odds of having an IPI increased twice for unemployed women (cOR 2.03; 95% CI 1.28–3.19; *p* = 0.002). Women with a parity of 5 or above had 4.19 times higher odds of having an IPI during pregnancy (95% CI 1.49–11.79; *p* = 0.006) than nulliparous women. Women with 4 to 7 visits and those with 8 or more ANC visits were 68% (cOR 0.32; 95% CI 0.11–0.89) and 63% (cOR 0.37; 95% CI 1.13–1.04) less likely to have an IPI when compared to those with 1 to 3 ANC visits, with a statistical significance of *p* = 0.030 and *p* = 0.060, respectively.

### 3.6. Factors Associated with IPIs in Pregnant Women: Multivariable Analysis

The results obtained from the multivariable logistic regression model are also depicted in Table 5, showing the adjusted association strength between the factors that remained in the final model and intestinal parasite infections in pregnant women. Results indicate that women with primary education, not working and with a parity of 5 or above, are more likely to have an IPI during pregnancy.

## 4. Discussion

Women living in LMICs are known to have a higher risk of acquiring an IPI during their pregnancy and consequently suffering complications from these NTDs [1]. This study aimed to identify the prevalence, treatments, and associated factors among pregnant women with an intestinal parasitic infection. This study confirms a high prevalence of intestinal parasitic infection among pregnant women in Sao Tome and Principe, as the overall prevalence was 58.2% (95% CI 52.9 to 63.3). Our prevalence is higher than that in nearby countries, namely, Ghana with 23.0% [33, 34] and Nigeria with 20.8% [35], but lower than that in other countries, such as Ethiopia, where it reaches 70.6% [36–38].

Soil-transmitted helminths (STHs) or geohelminths were the main IPI group among pregnant women enrolled in our study, in contrast to Ghana, which reports higher rates of intestinal protozoans [34].

Although higher in our study (90.9%), other studies from endemic countries also found a preponderance of *Ascaris lumbricoides* among pregnant women, such as Venezuela with 57% [13], Ethiopia with 32.7% [36], and Kenya with 6.5% [39]. In STP, previous data concerning IPI among children reported up to an 86.7% rate of infection, mainly with *Ascaris lumbricoides* (56.3%) and *Trichuris trichiura* (52.5%), reinforcing a previously existing high burden of helminthiasis in the country [38]. Therefore, these high rates of ascariasis and trichuriasis in STP illustrate that transmission occurs due to soil and domestic water supply with fecal pollution around homes with poor sanitation and improper sewage disposal [39].

In contrast, a very low rate of hookworm (1.4%) and strongyloidiasis (0.5%) in comparison to other studies [37, 38] was found, probably due to infrequently walking barefoot among the adult population in the country preventing larvae penetration in the feet skin [1, 37].

Regarding schistosomiasis, we found a 3% prevalence of *S intercalatum*. Overall estimates report that 10 million women in Africa per year have schistosomiasis during pregnancy although we could not find any study regarding *S intercalatum* infection in pregnant women in literature [40, 41]. This paucity of data can be related to the fact that *S intercalatum* is restricted to a few central African countries, is transmitted by *Bulinus forskalii*, and has a mild pathogenicity linked to rectal schistosomiasis and minor liver pathology [32, 42]. We observed that most pregnant women from our study with an *S intercalatum* infection had an urban residence. This “urbanization” of *S intercalatum* was previously reported in another study from central Africa [43]. This “urban” transmission of schistosoma occurs while walking through flooded streets (temporary snail breeding sites) instead of the traditional transmission in rural areas when people become infected through contact with parasite-harboring snails in natural water sources during activities such as fishing, farming, or swimming [44].

Concerning *Entamoeba histolytica*, a lower rate of 1.9% was found in this study compared to other settings, such as Venezuela, with a 12% rate [13]. Other intestinal protozoa, such as *Giardia duodenalis*, were also very uncommon (0.9%) in our study, perhaps due to methodological limitations in their identification since a previous study in the country, using molecular methods (PCR), found a 7.5% prevalence of *Giardia duodenalis* among children attending HAM [45].

Looking into the associated factors of intestinal parasitic infection, we identified that education, parity, and employment were significantly associated with IPI among pregnant women in this study. Primary education increased the odds of IPI in pregnant women, in accordance with other studies from Ethiopia [37] and Kenya [10], since better education is related to enhanced health-seeking behavior. The odds of IPI were almost twofold higher in unemployed pregnant women than in those employed. This relationship between unemployment and IPIs was already reported in other studies, reinforcing that a low economic standard, typically associated with unemployment, promotes IPIs in endemic settings [46, 47].

The odds of IPI were approximately four times higher among pregnant women with a parity of five or above, consistent with other studies that reported that age and parity were possible risk factors for parasitosis [1]. For instance, ascariasis in pregnancy was found to be most common in women between 20 and 29 years of age, and the prevalence increased with parity [1], similar to our study. In contrast, other authors state that multiparous women had reduced odds of IPIs compared to nulliparous women with the rationale that they might have experienced more ANC education on how to avoid IPIs in their previous pregnancies [33]. Our findings suggest a child-driven intrafamily transmission of parasites in STP since grand multiparous women have more children, which is in accordance with previous studies that described a high burden of IPI among children in STP [48].

Open defecation and not having access to improved water were associated with an IPI higher risk although no statistically significant difference was found. While the findings are in line with those reported from studies in Ghana [34], Colombia [49], and Mexico [50], they are contrary to those from Ethiopia [37], where the unavailability of toilet facilities was found to be significantly associated with IPI in pregnancy. Beyond this lack of association, we identified that more than 40% of all participants in this study reported having a daily practice of open defecation. Thus, Sao Tome and Principe will surely benefit if it takes the first step on the “sanitation ladder” proposed by the World Health Organization Program for Water Supply and Sanitation toward better health for all in the country [25].

Our analysis regarding anthelmintic prescription to infected pregnant women highlights that most were not treated at all, adverting to important “treatment missing opportunities.” This can be due to issues related to anthelmintic safety, namely, fear of teratogenic effects by health professionals and pregnant women, a significant obstacle also reported in other endemic countries [51].

Additionally, this study also reveals the high proportion of inadequate anthelmintic prescriptions. Regarding STH infection, most pregnant women received the recommended drugs (albendazole or mebendazole) although some still received metronidazole, which is specific to protozoan infection [1]. None of the pregnant women with *S intercalatum* infection took the recommended praziquantel (40 mg/kg), and two women were even inadequately treated with piperazine [20, 32]. Similarly, pregnant women infected with *E histolytica* were not treated, even though amebiasis treatment should be warranted even in asymptomatic carriers, not only because of the potential of developing the invasive disease but also to diminish the spread of disease [7, 52, 53].

The “treatment missing opportunities” identified by this study should be urgently addressed, first by training health professionals on proper prescription, and second, in case of pregnant women refusal of treatment during the pregnancy period, they should be referred and followed up for adequate treatment in the postpartum period.

As this is the first study in Sao Tome and Principe to provide comprehensive data concerning the burden of intestinal parasites among pregnant women, recommendations from the study will assist government health officials in policy development. Public health education and awareness campaigns combined with health professionals' education programs would also enhance women's knowledge of IPI prevention and ensure adequate anthelmintic therapeutic practices by health professionals.

### 4.1. Limitations

Although the study was conducted at a referral center (HAM) which serves most pregnant women in Sao Tome and Principe, its findings cannot be generalizable to other areas. Rural antenatal care services in STP may have a higher prevalence of IPIs among pregnant women since they are more exposed to potential sources of infection, such as contaminated water, farm animals, and wildlife [45]. In addition, they may also have distinct behavior and poorer hygiene practices and sanitation, increasing their risk of infection in comparison with urban pregnant women [45].

## 5. Conclusions

Intestinal parasitic infections are a high burden for pregnant women in Sao Tome and Principe, mainly for those with primary education, unemployed, and grand multiparous. Missing opportunities for IPI treatment, mainly for *S intercalatum* and *E histolytica*, should be addressed with health professionals' training and through the follow-up of women who refuse anthelmintic drugs during pregnancy for later postpartum treatment.

## Figures and Tables

**Table 1:** Baseline characteristics of the study sample (361), pregnant women with IPI (210), no-IPI (151), and associations with maternal characteristics.

	Total pregnant women (*n* = 361)	IPI (*n* = 210)	no-IPI (*n* = 151)	*p* value^*∗*^
Age				0.552
14–19	65 (18)	39 (18.6)	26 (17.2)	
20–34	233 (64.5)	131 (62.4)	102 (67.5)	
≥35	63 (17.5)	40 (19)	23 (15.2)	
Education				**0.002**
None	12 (3.3)	5 (2.4)	7 (4.6)	
Primary	196 (54.3)	132 (62.9)	64 (42.4)	
Secondary	126 (34.9)	61 (29)	65 (43)	
Higher	27 (7.5)	12 (5.7)	15 (9.9)	
Employment				**0.002**
Not working	250 (69.6)	158 (76)	92 (60.9)	
Working	109 (30.4)	50 (24)	59 (39.1)	
Marital status				0.721
Union/married	303 (85.4)	177 (85.9)	126 (84.6)	
Single	52 (14.6)	29 (14.1)	23 (15.4)	
Baby's father education				0.485^#^
None	8 (3.3)	3 (2.1)	5 (5)	
Primary	116 (47.3)	71 (49)	45 (45)	
Secondary	94 (38.4)	57 (39.3)	37 (37)	
Higher	27 (11)	14 (9.7)	13 (13)	
Residence				0.089
Urban	166 (46.4)	120 (57.4)	72 (48.3)	
Rural	192 (53.6)	89 (42.6)	77 (51.7)	
Water source				0.261
Improved water	304 (84.2)	173 (82.4)	131 (86.8)	
No	57 (15.8)	37 (17.6)	20 (13.2)	
Household sanitation				0.247
With sanitation	202 (56.4)	112 (53.8)	60 (90)	
Open defecation	156 (43.6)	96 (46.2)	40 (60)	
Parity				**0.010**
0	103 (28.5)	57 (27.1)	46 (30.5)	
1–4	227 (62.9)	127 (60.5)	100 (66.2)	
5+	31 (8.6)	26 (12.4)	5 (3.3)	
ANC visits				0.075
<4	24 (6.6)	19 (9)	5 (3.3)	
4–7	168 (46.5)	92 (43.8)	76 (50.3)	
≥8	169 (46.8)	99 (47.1)	70 (46.4)	

ANC: antenatal care and IPI: intestinal parasitic infection. *p* value <0.05 marked as bold text. ^*∗*^*p* value calculated from chi-square test.^#^*p* value calculated from Fisher's exact test.

**Table 2:** Parasites identified in the 210 positive copro-parasitological exams.

	Stool specimens	Frequency (*n*=)	Percentage^*∗*^ (%)
Helminths	*Ascaris lumbricoides*	191	90.9
	*Trichuris trichiura*	29	13.8
	*Ancylostoma duodenale*	3	1.4
	*Strongyloides stercoralis*	1	0.5

Schistosoma	*Schistosoma intercalatum*	11	5.2

Intestinal protozoans	Pathogenic		
	*Entamoeba histolytica*	7	3.3
	*Giardia duodenalis*	2	0.9
	Nonpathogenic		
	*Endolimax nana*	1	0.5

^*∗*^parasite individual percentages add more than 100% due to copro-parasitological exams with more than one parasite per sample. Out of the 210 exams analyzed, a total of 245 different parasite specimens were identified.

**Table 3:** Polyparasitic infections (coinfection of two or more parasite species in the same pregnant women).

Polyparasitism	Frequency (*n*=)	Percentage^*∗*^ (%)
*Ascaris lumbricoides* + *Trichuris trichiura*	18	
*Schistosoma intercalatum* + *Entamoeba histolytica*	3	
*Ascaris lumbricoides* *+* *Ancylostoma duodenale*	2	
*Ascaris lumbricoides* + *Schistosoma intercalatum*	2	
TOTAL	25	11.9

^*∗*^From the 210 pregnant women with an IPI, a total of 25 (11.9%) had two different parasites identified in their copro-parasitologial exam. The helminths group total 224 including polyparasitic infections (Table 2), but only 202 pregnant women had helminthic monoparasitic infection.

**Table 4:** ANC anthelminthics prescription registered in the ANC pregnancy cards.

IPIs treatment	Helminths (*n* = 202)^*∗*^	*S intercalatum* (*n* = 11)^*∗*^	*E histolytica* (*n* = 7)^*∗*^
No	147 (72.7)	8 (72.8)	4 (57.1)
Missing	9 (4.5)	1 (9.0)	0
Yes	46 (22.8)	2 (18.2)	3 (42.9)
Anthelminthic drug			
Albendazole	20 (43.5)	0	1 (33.3)
Mebendazole	17 (37)	0	2 (66.7)
Metronidazole	6 (13)	0	0
Piperazine	3 (6.5)	2 (100)	0
Adequate prescription	40 (87)	0	0
Inadequate prescription	6 (13)	2 (100)	3 (100)

IPI: intestinal parasitic infection. ^*∗*^For the anthelmintic prescription analysis, three different groups were considered since the recommended drugs are different for helminthiasis (albendazole, mebendazole, or piperazine), schistosomiasis (praziquantel) [20, 32], and amebiasis (paromomycin, nitroimidazoles as metronidazole or tinidazole) [7, 8]. The sum refers to 220 infections: 202 monoparasitic helminthiasis plus 11 schistosomiasis (*S intercalatum*), and 7 amebiasis (*E histolytica*). Schistosomiasis and amebiasis coinfection cases were included. Giardiasis cases (2) were not described due to missing information on anthelmintic prescription.

**Table 5:** Results of univariable and multivariable logistic regression analysis regarding IPI associated factors.

	IPI (*n* = 210)	no-IPI (*n* = 151)	cOR (95% CI)	*p* value	AOR (95% CI)
Age					
14–19	39 (18.6)	17.2 (26)	1		
20–34	131 (62.4)	67.5 (102)	0.86 (0.48–1.49)	0.587	
≥35	40 (19)	15.2 (23)	1.16 (0.57–2.37)	0.685	
Education					
None	5 (2.4)	4.6 (7)	0.89 (0.23–3.53)	0.872	1.73 (1.10–22.71)^*∗*^
Primary	132 (62.9)	42.4 (64)	2.58 (1.14–5.83)	**0.023**	
Secondary	61 (29)	43 (65)	1.17 (0.51–2.71)	0.708	
Higher	12 (5.7)	9.9 (15)	1		
Employment					
Not working	158 (76)	60.9 (92)	2.03 (1.28–3.19)	**0.002**	1.94 (1.20–3.13)^*∗*^,^*∗*^
Working	50 (24)	39.1 (59)	1		
Marital status					
Union/married	177 (85.9)	84.6 (126)	1		
Single	29 (14.1)	15.4 (23)	0.89 (0.49–1.62)	0.721	
Baby's father education					
None	3 (2.1)	5 (5)	0.56 (0.11–2.81)	0.479	
Primary	71 (49)	45 (45)	1.46 (0.63–3.40)	0.374	
Secondary	57 (39.3)	37 (37)	1.43 (0.60–3.38)	0.415	
Higher	14 (9.7)	13 (13)	1		
Residence					
Urban	120 (57.4)	72 (48.3)	1		
Rural	89 (42.6)	77 (51.7)	1.44 (0.95–2.2)	0.089	
Water source					
Improved water	173 (82.4)	131 (86.8)	1		
No	37 (17.6)	20 (13.2)	1.40 (0.77–2.53)	0.262	
Household sanitation					
Sanitation	112 (53.8)	60 (90)	1		
Open defecation	96 (46.2)	40 (60)	1.29 (0.84–1.97)	0.247	
Parity					
0	57 (27.1)	46 (30.5)	1		
1–4	127 (60.5)	100 (66.2)	1.03 (0.64–1.64)	0.918	
5+	26 (12.4)	5 (3.3)	4.19 (1.49–11.79)	**0.006**	3.82 (1.32–11.08)^*∗*^
*ANC visits*					
<4	19 (9)	5 (3.3)	1		
4–7	92 (43.8)	76 (50.3)	0.32 (0.11–0.89)	**0.030**	
≥8	99 (47.1)	70 (46.4)	0.37 (0.13–1.04)	**0.060**	

*p* value <0.05 marked as bold text; ^*∗*^at a *p* value <0.02, and ^*∗*^, ^*∗*^ at a *p* value <0.001. IPI: intestinal parasitic infection.

## Data Availability

The datasets used and/or analyzed during the current study are all included within the manuscript itself.

## Abbreviations

STP: Sao Tome and Principe
HAM: Hospital Dr. Ayres de Menezes
OR: Odds ratio
CI: Confidence interval
ANC: Antenatal care
WHO: World Health Organization
NTD: Neglected tropical diseases
LBW: Low birth weight.
